# Physicians’ engagement: Medical Care Groups

**DOI:** 10.1590/S1679-45082016ED3757

**Published:** 2016

**Authors:** Sidney Klajner

**Affiliations:** 1 Sociedade Beneficente Israelita Brasileira Albert Einstein, São Paulo, SP, Brazil; Hospital Israelita Albert Einstein, São Paulo, SP, Brazil.

## CLINICAL STAFF PROJECT

### Introduction

The relation between the *Hospital Albert Einstein* and its clinical staff dates from the start of its activities. The invitation to physicians to work at the hospital, made by the President himself, an initial model of attracting doctors, was followed by the orchestration of the form of registering professionals, when the need for confirmation of qualification and skills arose, until reaching the current model in which, besides credentials, the physician is accompanied and his/her practice is monitored.

During this journey of more than 40 years, the context in which the medical activity has developed, both in and out of the hospital, immensely influenced the form of organization of our physicians. Additionally, the doctor-patient relationship itself was an influence on the activity and relationship among physicians, since we have gone from a patronizing posture exerted by the physician, who used to judge alone what should or should not be a part of the treatment, to a situation in which the power of decision is shared with the patient. In fact, today access to information is easier due to the Internet, by means of sources of information, patient communities, social media, mobile devices, among others. A recent proof of this was the partnership between the *Hospital Albert Einstein* and Google, to provide relevant health information to internet users in their search.

More complex Medicine, with superspecialization of physicians and of the multiprofessional team; the emerging technological devices that are increasingly growing and adding value to treatment outcome; the need for interaction among different specialties to provide a better outcome for patients; and mainly, the added cost of diagnosis and treatment, resulting from more professionals, physical and technological structures – all these have currently obliged us to prepare for a near future, in which there will be no room for “avoidable” complications, waste of resources, remuneration mechanisms based on the use of materials and medications. There will no longer be a way to carry out our mission, of excellence in care, without offering the best available to our patients.

In face of the challenges for the future of our organization, we conclude that there is no way to conduct the best medical activity without having an extremely organized and engaged clinical staff, aligned with the best interests of the organization and in favor of the patient. To this end, we must organize our physicians differently, attracting and training those who are younger, adding knowledge and team-work conditions, with the participation of other healthcare professional, developing the leadership to organize care delivered by physicians, who are now informed and capable of playing this role in the context of 21^st^ century Medicine.

### History

The *Hospital Albert Einstein* began its activities in 1971, and since then, the most significant facts surrounding its clinical staff may be described as follows.

Since the visionary dream of Manoel Tabacow Hidal and the foundation of the *Sociedade*, in 1955, the attraction of physicians occurred by invitation from its presidents: Dr. Hidal himself, who perceived the possibility of creating a structure of departments similar to that of Academy, and later, Dr. Josef Fehér, who began to attract and invite physicians based on a model of technological acquisition never seen before in hospitals of our country.

Few doctors already established as university professors or seniors with activities in the central region of the city came to work at the hospital, due to the distance to the Morumbi area and the fact that this was a new hospital. This motivated the coming of young physicians, who saw the *Hospital Albert Einstein* as a means of “planting” their careers.

At the end of Dr. Josef Fehér’s term in office, and especially during the administration of Dr. Reynaldo Brandt, a movement for quality based on metrics, and not on reputation, processes, and accreditations began. The participation of several doctors of the clinical staff was crucial for us to obtain, in 1999, the first accreditation from the Joint Commission International.

It was in the 1990’s that the professionalization of some physicians began in areas to provide more structured support to the activities of the organization, including the emergency department, intensive care unit, laboratory, and imaging.

In 1996, with the increased number of hired physicians, systemization of emergency care (Advanced Cardiovascular Life Support − ACLS, Advanced Trauma Life Support^®^ − ATLS, and Pediatric Advanced Life Support − PALS) and support groups formed by non-hired clinical staff physicians, we noted a more evenly distributed “medical leadership” in the different areas. This is when the Formal Clinical Board was established, which is today a requirement of the Federal Council of Medicine, and was led for the first time by Dr. Nelson Hamerschlak.

This initial organization enabled choosing physicians to act as support and background in the emergency department, contributed towards the strengthening of physicians and clinical staff, and led to the creation of a medical indicator. Additionally, the first medical committees were established, besides the Continuing Medical Education program and the specialty forums. In the year 2000, the *Instituto Israelita de Ensino e Pesquisa* was born in the form in which it exists today, developed from the study center that already existed.

In 2003, the society’s management became professional with the training of leadership, managing physicians, and with this, it weakened the clinical staff and its decision-making power. The change in principles of this administration, represented by its CEO, after external consultancy, contributed towards a series of changes in the form of remuneration, salary cuts, and dismissals that generated a large impact on the work environment.

Also created were strategic programs, such as the Medical Relationship Program, which is increasingly more sophisticated until today, always with meritocracy as its basis. This program included feedback, quality goals, and the patient safety program.

The beginning of government partnerships should also be mentioned, with activity in the Outpatient Medical Care units (AMA) and the Family Health Program (PSF) at the *Hospital Municipal Dr. Moysés Deutsch - M’Boi Mirim*.

At the end of the 2000 decade, part of the Clinical Board attributes were transferred to the institutional Medical Practice Board, such as the revision of the medical indicator and backup, the occupation of the Vicky and Joseph Safra Pavilion medical offices, and other benefits and privileges based on meritocracy.

The historical analysis of the relation between the *Sociedade* and its physicians allowed drawing some conclusions, which serve as teachings and guidance in formulating a new relationship model capable of maintaining the position of medical leadership that we occupy until today.

### Assumptions for the project

– Exponential growth of the *Sociedade*, in all areas (care, teaching, and research), transforming it into a true health system.

– More complex Medicine demands team work, with interaction between the specialist, multidisciplinary team, areas, or services. It also requires cooperation among physicians, instead of mere internal competition. The quality of the relationship among physicians should evolve.

– A need for a new mindset, with a systemic view by physicians, which includes notions of management, health economics, resources, quality and safety, as well as new concepts about ethics.

– Expansion of the teaching activities.

– Need to attract young talent and the capacity for coaching in face of the ageing of the current clinical staff and the lack of motivation of hired physicians due to lack of opportunities for growth. Creating opportunity for Careers in Care, Teaching, Research, and Social Responsibility.

– Strategic partnerships with renowned international institutions.

– Expansion of institutional patients.

– Threat of the influence of healthcare insurance plans in medical activity by means of incentives, and the vertical focus on healthcare services.

– Threat of the influence of the industry of supplies and medications in patient safety, with the risk of shaking the solid pillars of medical and institutional ethics.

– Support for training physicians within the *Hospital Albert Einstein* (medical school, medical residency, graduate studies, and international fellowships).

– Added value to the doctor-patient relationship in medical care (patient experience).

– Maturing of the meritocracy indicators in the relation with the organization (physician engagement).

### Vision and guidelines

The vision is based on being a model that attracts, integrates into a multidisciplinary structure, develops, and fosters loyalty of the clinical staff of *Hospital Albert Einstein*, for excellence in care to their patients.

The 13 central strategic guidelines, which are no more than what we desire for the future of our hospital, are the following:

1. The doctor-patient relationship (patients first).
2. To map and develop networks of professional relationships within the clinical staff.
3. To establish a new model of medical leadership, based on intense participation of the physician and considering the practice.
4. To organize the relations and activities of the clinical staff by similar interests.
5. To improve the technological and administrative support to the medical practice and development.
6. To offer the clinical staff support to conduct clinical and translational research, and share the results.
7. To assure continued medical education.
8. To offer opportunities for development of an amplified vision of health-related activities (education).
9. To actively support the development of a transdisciplinary approach in medical practice, involving diverse areas, services, and professionals.
10. To create a flexible model of a medical career, as a means of attracting talents.
11. To foster ongoing improvement of the model of relationship with the clinical staff.
12. To seek full satisfaction of the physician with his/her career and professional life at the hospital.
13. To seek attractive remuneration that is aligned with the interests of society.

### Strategy

Initially, we decided to choose three of the 13 strategic guidelines so that we could, in a practical manner, truly initiate the project: (1) returning to physicians the leadership of their practice; (2) organizing the clinical staff according to similar interests, not to specialties; (3) establishing a flexible model of medical career. The form found by the physicians to organize these guidelines was by means of the Medical Care Groups (GMA - *Grupos Médicos Assistenciais*).

The GMA is a group of care professionals organized by the Quality and Care Committee of the Board, responsible for the processes and actions geared towards improving the quality and safety of patient care given. These groups are established based on specific diseases and conditions, or on therapies and technologies.

The objectives of the GMA are:

– To align their activities with the ethical principles and strategy of the organization.

– To promote compliance with evidence-based practices.

– To develop care focused on a multidisciplinary team.

– To develop patient-centered care.

– To propose improvements in technological and administrative support to the medical practice and development.

– To establish methods and opportunities for learning.

– To develop teaching and research.

– To increase access of patients to the organization services.

– To give technical support within its active realm for the organization’s activities along with the regulating agencies, financial sources, and healthcare service providers.

– To participate in processes of evaluation of outcomes, patient satisfaction, and efficiency.

– To identify, attract, retain, and develop medical talents.

– To develop professional relationship networks within the clinical staff, for continuity of treatment and maintenance of the patient in the Einstein system.

– To propose new services, remuneration models, and levels of fees.

With the organization of the clinical staff into the GMA and its mature performance, in synergy with the management executive professionals, we intend to encourage, within its activities, the ten remaining guidelines previously described.

### Current phase

Three pilot GMAs were created to start the work. Clinical staff physicians from several specialties - both autonomous and hired, were invited, together with members of the multiprofessional team. The invitations were based on the Segmentation Program. The groups were Metabolic Syndrome, Hepatology, and Endovascular Interventions.

The activities of these groups allowed understanding and polishing the *modus operandi* of each one of them. Heated discussions were initiated and all of them led to working decisions and proposals with an impact on medical practice, teaching (symposia), and research. Additionally, there was participation and total engagement of the multidisciplinary team.

The experience acquired with the pilot GMAs spawned the expansion in the number of groups, and we chose to transform into GMA the groups that already worked within the strategic specialty programs (cardiology, oncology, neurology, orthopedics, transplants, and surgery). Today there are 29 fully active GMA, and among these, to date, 55 macro-actions have arisen related to improvement of patient care, of the population’s health focused on prevention and early identification of risks, and of cost and waste reduction. These three pillars of quality make up the policy we adopted, which was proposed by the Institute for Healthcare Improvement*,* a strategic partner of the *Hospital Albert Einstein*. Added to these results are intangible benefits, such as sharing knowledge and a better relationship among the clinical staff and between physicians and the multidisciplinary teams.

The GMA coordinators, elected by the group, are prepared for their activity in training course modules for coordinators. The modules are organized the Medical Board, and they attempt to close all gaps in basic training, especially related to management, interpersonal relationships, and governance of the organization. Periodically, these coordinators participate in the Quality and Care Committee meetings to present their most significant achievements and plans of their respective GMA, as well as to request support from the organization for projects that demand funds or important changes in some organizational process or practice.

The GMA have also proven to be a strategic initiative for attracting and retaining young talents, who are exposed to complex discussions in a collegiate environment, without the weight of hierarchy. The more experienced clinical staff, on the other hand, can exert its capacity of coaching and keep abreast with the ideas and new technologies proposed by all types of professionals. The indicators and cases of standardization, the improvement of processes and the already implemented innovations show that the GMA are an important organizational strategy in the ongoing search for higher levels of care to patients. Finally, the GMA have also become a relevant component for attracting, integrating, and creating loyalty in the clinical staff, since they are aligned with the meritocratic standards of our organization.

